# Mild chronic cerebral hypoperfusion induces neurovascular dysfunction, triggering peripheral beta-amyloid brain entry and aggregation

**DOI:** 10.1186/2051-5960-1-75

**Published:** 2013-11-13

**Authors:** Ayman ElAli, Peter Thériault, Paul Préfontaine, Serge Rivest

**Affiliations:** 1 Neuroscience Laboratory, CHU de Québec Research Center and Department of Molecular Medicine, Faculty of Medicine, Laval University, 2705 Laurier boul., Québec City, QC G1V 4G2, Canada

**Keywords:** Alzheimer’s disease, Neurovascular unit, Blood–brain barrier, Mild chronic cerebral hypoperfusion, Beta-amyloid, Signal transduction

## Abstract

**Background:**

The Blood–brain barrier (BBB) controls brain supply with oxygen and nutrients, and protects the brain from toxic metabolites, such as beta-amyloid (Aβ) peptides. The neurovascular unit (NVU) couples vascular and neuronal functions by controlling BBB parameters based on brain needs. As such, NVU/BBB dysfunction, associated to irregularities in cerebral blood flow (CBF), has been proposed to contribute in the pathogenesis of Alzheimer’s disease (AD), mainly by impairing cerebral Aβ clearance. However, the spatiotemporal contribution of the NVU/BBB in the neurodegenerative cascades remains elusive.

**Results:**

By using C57BL/6J mice subjected to right common carotid artery (rCCA) permanent ligation in order to induce mild chronic cerebral hypoperfusion, we show here that cerebral hypoperfusion induced NVU dysfunction by reducing ABCB1 protein expression in brain capillaries. ABCB1 reduction was mainly triggered by an enhanced Glycogen Synthase Kinase 3 (GSK3β) activation, which decreased β-catenin nuclear abundance. Moreover, cerebral hypoperfusion triggered early vascular deposition of peripherally applied human Aβ_1-42_ peptides, which has shifted from highly vascular to the parenchyma 6 weeks later, forming small stable Aβ deposits. Hypoperfusion induced a deregulation in glucose metabolism, as brain reperfusion, or the administration of a high dose of glucose, diminished GSK3β activation, recuperated β-catenin nuclear abundance, reestablished ABCB1 protein expression, and prevented Aβ vascular early deposition. These results demonstrate that mild chronic cerebral hypoperfusion creates a metabolically deregulated microenvironment, thus triggering the brain entry and aggregation of peripherally applied human Aβ_1-42_ peptides.

**Conclusion:**

Our study offers new insights on the initiation of the neurodegenerative cascades observed in AD, which could be valuable in developing adequate treatment strategies.

## Background

Amyloid deposits and neurofibrillary tangles formation are the core pathological hallmarks of Alzheimer's disease (AD) [1]. The clinical manifestations of AD begin with mild memory deficits that evolve with time to reach severe cognitive impairment and loss of executive functions [2]. Despite the extensive research, the exact causes leading to the initiation of the neurodegenerative cascades in AD remain not fully understood. Nonetheless, beta-amyloid (Aβ) peptides seem to play central role. Amyloid Precursor Protein (APP) sequential proteolytic cleavage generates small peptides, among which are Aβ_1–40_ and Aβ_1–42_[3], which oligomerize to form soluble oligomers, and further aggregate into insoluble amyloid deposits, leading to the formation of Aβ plaques [4]. Although the link between Aβ plaques and the cognitive decline observed in AD is still elusive, the toxicity of soluble Aβ oligomers in the brain of either AD patients [5], or mouse models of AD [6] has been demonstrated. Brain levels of soluble Aβ oligomers play detrimental role in AD pathogenesis, by inducing neuronal dysfunction that have been shown to take place even before neurodegenerative cascades [4].

In contrast to familial AD that is provoked by genetic mutations in genes related to Aβ processing, sporadic AD is caused by environmental heterogeneous risk factors and accounts for the majority of AD cases [7]. Among these risk factors, blood pressure, heart disease, cerebrovascular diseases, diabetes and high blood cholesterol, play crucial role, by deeply affecting brain perfusion and vascular integrity [8]. The brain consumes 20% of nutrients - mainly glucose - and oxygen present in the blood [9]. Neurons are highly vulnerable cells that rely on brain’s highly dynamic and complex vascular network to assure accurate and adequate distribution of oxygen and glucose, which are deeply affected by cerebral blood supply controlled by cerebral blood flow (CBF) [8]. A reduction in CBF, at least induces rapid neuronal stress, which can evolve to an irreversible damage if this reduction persisted and/or is greater than 80% [10]. Therefore, optimal brain perfusion and cerebrovascular integrity are essential for proper brain function. Indeed, impairments in both CBF and glucose metabolism have been reported in AD patients [11,12], and in mouse models of AD [13]. More interestingly, deregulations in cerebrovascular perfusion have been suggested to take place even before cognitive decline [14].

The blood–brain barrier (BBB) plays a central role in controlling the exchange of oxygen and nutrients [8,15]. The BBB is formed by tightly sealed endothelial cells, constituting a non permissive physical barrier that separates the blood from the brain. The BBB controls brain homeostasis by adjusting oxygen and nutrients delivery based on brain needs, and by removing toxic compounds from the brain [16]. Brain endothelial cells actively interact with extracellular matrix proteins that constitute the perivascular space, pericytes, astrocytes, microglia and neurons, forming the neurovascular unit (NVU) that couples vascular and neuronal functions by controlling BBB parameters [15]. The NVU is complemented by sophisticated transport systems, which allow continuous and precise control of brain homeostasis, among which are the ATP-Binding Cassette (ABC) transporter family, namely ABCB1 that contributes in brain detoxification by removing toxic compounds, among which are Aβ peptides [17]. Interestingly, ABCB1 function and expression have been reported to be impaired in AD [18,19].

Recently, the two-hit vascular hypothesis incorporated cerebrovascular dysfunction as a major player in AD pathogenesis [8,9]. Such a hypothesis suggests that several risk factors affecting vascular functions are involved in AD pathogenesis. These include hypertension, diabetes, heart disease and cerebrovascular diseases, which affect NVU function and lead to an excessive accumulation of Aβ in brain parenchyma due to an impaired clearance. The neuronal dysfunction may also be caused by the presence of metabolically distressed microenvironment due to a chronic cerebral hypoperfusion (i.e. oligaemia). As such, in the present study, we aimed to investigate the effects of mild chronic cerebral hypoperfusion on NVU function and Aβ deposition. For this purpose, we used C57BL/6J mice subjected to right common carotid artery (rCCA) permanent ligation (i.e. one vessel occlusion = 1 VO) in order to induce mild chronic cerebral hypoperfusion [20]. Mice were intravenously injected with either saline or human Aβ_1-42_ peptides. The molecular mechanisms involved in hypoperfusion-induced NVU dysfunction were deciphered, and Aβ_1-42_ peptides brain entry and aggregation upon hypoperfusion were investigated.

Here we report that mild chronic cerebral hypoperfusion induces a chronic dysfunction of the NVU, triggering early vascular deposition of systemically injected Aβ_1-42_ peptides, which has also shifted to the parenchyma 6 weeks later, thus potentially seeding the brain. These alterations were essentially prevented by brain reperfusion or the administration of a high dose of glucose, indicating that glucose metabolism deregulation at the NVU upon hypoperfusion constitutes a major player in Aβ homeostasis.

## Methods

### Animal groups

Experiments were performed according to the Canadian Council on Animal Care guidelines, as administered by the Laval University Animal Welfare Committee. All efforts were made to reduce the number of animals used and to avoid their suffering. C57BL/6J mice (20–25 g) were housed and acclimated to standard laboratory conditions (12-hour light/dark cycle / lights on at 7:00 AM and off at 7:00 PM) with free access to chow and water. Mice were subjected to one vessel occlusion (i.e. 1 VO) and sacrified as described in (Additional file 1: Figure S1).

For molecular analysis, mice were perfused with saline (0.9% NaCl), brains were removed and immediately frozen in dry ice (Additional file 1: Figure S1; Protocol A). For immunofluorescence, and Thioflavin S analysis, mice were perfused with 4% paraformaldehyde (PFA) in 0.1 M phosphate buffer, brains were removed and postfixed in 4% PFA (pH 7.4) at 4°C and then immersed in a PFA solution containing 10% sucrose overnight at 4°C. Fixed brains were frozen with dry ice/ethanol mixture, mounted on a microtome (Leica) and cut into 25 μm coronal sections. The collected slices were placed in tissue cryoprotectant solution containing 0.05 M sodium phosphate buffer (pH 7.3), 30% ethylene glycol, and 20% glycerol, and stored at −20°C until analysis (Additional file 1: Figure S1; Protocol B).

### Aβ_1–42_ solution preparation

Monomeric HiLyte Fluor 555-labeled Aβ_1–42_, and non labelled Aβ_1–42_ (Anaspec, Fremont, CA, USA) were prepared as previously described [21]. Briefly, lyophilized labelled, or non labelled Aβ_1–42_, was dissolved in 1,1,1,3,3,3-hexafluoro-2-propanol (Sigma-Aldrich, St. Louis, MO, USA) to 1 μg/μl, dried under vacuum, and stored at −80°C. Immediately before injection, Aβ_1–42_ was dissolved in DMSO (Sigma-Aldrich) to 5 μg/μl, and was diluted in 0.9% NaCl sterile solution to obtain final dilutions of either 0.25 ng/μl (functional studies), or 0.1 μg/μl (proof of concept studies), which were always freshly prepared to avoid peptide’s oligomerization, and or degradation.

### Induction of mild chronic cerebral hypoperfusion

Four-month-old C57BL/6J mice were subjected to either sham surgery, or to 1 VO by permanently occluding the rCCA, under anesthesia with 2% isoflurane. Briefly, a midline cervical incision was made and the rCCA was exposed and double-ligated with 6–0 silk suture thread (1 VO groups). The sham surgery consisted of a midline cervical incision under isoflurane anesthesia (2%) followed by the exposition of the rCCA (sham group). In both groups, the skin incision was closed with interrupted 6–0 silk sutures and the animals were then placed on a temperature-controlled blanket until they recovered from anesthesia. Twenty-four hours after 1 VO, two groups of mice were re-operated to reperfuse rCCA and reestablish cerebrovascular perfusion or received single intravenous injection, via the tail vein, of high dose of glucose (5 mg/ml, i.e. 5x physiological concentration) (Additional file 1: Figure S1; Protocol C, D).

### Human Aβ_1-42_ injection upon mild chronic cerebral hypoperfusion

For imaging proof of concept studies (Additional file 1: Figure S1; Protocol E) hypoperfused animals received 10 μg of human Aβ_1-42_ Hilyte Fluor 555 intravenously via the tail vein, and Aβ was visualized by using two-photon intravital imaging in both contralateral, and ipsilateral hemispheres, 3 hours and 48 hours after injection. For functional studies (Additional file 1: Figure S1; Protocol F), hypoperfused animals received 25 ng of human Aβ_1-42_ Hilyte Fluor 555 intravenously via the tail vein, and Aβ was visualized by using two-photon intravital imaging in both contralateral, and ipsilateral hemispheres, 3 hours and 6 weeks after injection. In order to visualize Aβ deposits 6 weeks after Aβ_1-42_, Congo Red solution (4 μl; 1.5 μl/min) was injected in the Magna Cisterna 24 hours before imaging, to assure a complete distribution of the dye in the cerebrospinal fluid (CSF), and interstitial fluid (ISF). For molecular analysis studies, hypoperfused animals received 25 ng of non labeled Aβ_1-42_ intravenously via the tail vein, and were sacrified 24 hours after injection (Additional file 1: Figure S1; Protocol C).

### Two-photon intravital microscopy

The two-photon intravital microscopy procedure was performed as described previously [22]. Briefly, mice were deeply anesthetized with 2% isoflurane and mounted in a cranial stereotaxic apparatus (David Kopf Instruments, Tujunga, CA). An incision was made to expose the skull and two small cranial windows were drilled corresponding to the following coordinates: (i) A/P +0.83 mm, M/L +0.5 mm, and A/P +0.83 mm, M/L −0.5 mm relative to the bregma (Additional file 2: Figure S2). All images were acquired on an Olympus FV1000 MPE two-photon microscope dedicated to intravital imaging. The two-photon Mai Tai DeepSee laser (Spectra-Physics, Newport Corp., Santa Clara, CA) was tuned at 950 nm for all the experiments, and the output power was set between 14 and 84 mW. Brain tissues were imaged using an Olympus Ultra 25X MPE water immersion objective (1.05 NA), with filter set bandwidths optimized for CFP (460–500 nm), YFP (520–560 nm), Texas Red/DsRed (575–630 nm) and Qdot 705/800 (669–800 nm) imaging. Detector sensitivity and gain were set to achieve the optimal dynamic range of detection. Using the Olympus Fluoview software (version 3.0a), images with a resolution of 512 × 512 pixels were acquired at different zoom factors (1X to 3X) and at 2.5 frames per second with auto-Hv option enabled, and exported as 24-bit RGB TIF files, while metadata for subsequent automatic setting-detection were exported in a TXT file. Kalman filter was used during scanning in order to reduce background. For each brain hemisphere imaging, a total of 101 frames were acquired from the cortex at a depth ranging from 50 μm (i.e. cortex surface) to 250 μm (inside the cortex), for a total depth of 200 μm. Acquired images were either stacked for 2-dimensional illustrations, or processed using ImageJ image analysis software to produce 3-dimensional illustrations represented as video sections.

### Immunofluorescence staining

Free-floating sections were washed with potassium phosphate-buffered saline (KPBS) (3x, 10 minutes) and then incubated for 20 minutes in a permeabilization/blocking solution containing 4% goat serum, 1% bovine serum albumin (BSA) (Sigma-Aldrich), and 0.4% Triton X-100 (Sigma-Aldrich) in KPBS. Sections were incubated overnight at 4°C with different primary antibodies diluted in the same permeabilization/blocking solution. The following primary antibodies were used: mouse anti-human Aβ monoclonal antibody (1/1500) (6E10, Covance Inc., Princeton, NJ, USA), rat anti-mouse CD31 monoclonal antibody (1/500) (BD Pharmingen, Franklin Lakes, NJ, USA), and mouse anti-Glial Fibrillary Acidic Protein (1/1500) (GFAP) monoclonal antibody (GA5, Chemicon International, Temecula, CA, USA). Afterwards, the sections were rinsed in KPBS (3x, 10 minutes), followed by 2 hours incubation with Alexa Fluor 488-conjugated goat anti-mouse secondary antibody, Alexa Fluor 488-conjugated goat anti-rat secondary antibody, or Cy5--conjugated goat anti-mouse secondary antibody (Jackson ImmunoResearch Laboratories, West Grove, PA, USA). Sections were incubated overnight under light protected vacuum to allow an optimal fixation of brain sections on slides. The next day, sections were rinsed in KPBS (3x, 10 minutes), stained with 0.0002% DAPI for 5 minutes, rinsed again in KPBS (3×, 10 minutes), mounted onto SuperFrost slides (Fisher Scientific), and coverslipped with antifade medium composed of 96 mM Tris–HCl, pH 8.0, 24% glycerol, 9.6% polyvinyl alcohol, and 2.5% diazabicyclooctane (Sigma-Aldrich). Epifluorescence images were taken using a Nikon C80i microscope equipped with both a motorized stage (Ludl, Hawthorne, NY, USA) and a Microfire charge couple device color camera (Optronics, Goleta, CA, USA). Confocal laser scanning microscopy was performed with a BX-61 microscope equipped with the Fluoview SV500 imaging software 4.3 (Olympus America Inc., Melville, NY, USA).

### Thioflavin S staining

Free-floating sections were stained with 1% Thioflavin S as described previously [23] with small modifications. Briefly, sections were washed with KPBS (3×, 10 minutes) and then incubated for 8 minutes 1% Thioflavin S solution in distilled water. Sections were washed in 100% ethanol (1×, 1 minute), then in 80% ethanol/water (2×, 1 minute), then rinsed in distilled water (3×, 1 minute), placed in KPBS solution, and finally mounted onto SuperFrost slides, and coverslipped with antifade medium. For immunofluorescent staining combined with 1% Thioflavin S staining, the former was performed as described above, and the time of washing steps with ethanol in the latter were decreased (30 seconds instead of one minute) to not alter antibody-epitope bindings. Stereological analysis was performed as previously described [21]. The contours of the cortex, or hippocampus areas, of both contralateral and ipsilateral were traced as virtual overlay on the steamed images. The number of Thiofalvin S positive brain capillaries was counted in the total structure of five sections per brain (i.e. in all cortex, and hippocampus).

### Entrapped IgG in brain capillaries

Free-floating sections were washed with KPBS (3×, 10 minutes) and then incubated for 20 minutes in a permeabilization/blocking solution containing 4% goat serum, 1% BSA (Sigma-Aldrich), and 0.4% Triton X-100 in KPBS. For endogenous IgG detection, sections were incubated for 2 hours with Alexa Fluor 488-conjugated goat anti-mouse secondary antibody. Sections were rinsed in KPBS (3×, 10 minutes), stained with 0.0002% DAPI for 5 minutes, rinsed again in KPBS (3×, 10 minutes), mounted onto SuperFrost slides (Fisher Scientific), and coverslipped with antifade medium. The number of IgG positive brain capillaries was counted in the whole structure of the cortex, and the hippocampus separately. Stereological analysis was performed as described here above.

### Brain capillaries isolation

Brain capillaries from contralateral and ipsilateral hemispheres were isolated on dextran gradient as described previously [24]. Contralateral and ipsilateral hemispheres, of each animal, were separated and gently homogenized in a Teflon glass homogenizer in ice-cold microvessel (i.e. capillaries) isolation buffer (MIB; 15 mM Hepes, 147 mM NaCl, 4 mM KCl, 3 mM CaCl_2_, and 12 mM MgCl_2_) supplemented with 5% Protease Inhibitor Cocktail (P8340; Sigma) and 1% Phosphatase Inhibitor Cocktail 2 (P5726; Sigma). Homogenates were centrifuged at 1000 rcf for 10 min at 4°C. The resulting pellets were resuspended in 20% dextran (molecular weight, 64,000 to 76,000; D4751, Sigma) in MIB. Suspensions were centrifuged at 4400 rcf for 20 min at 4°C. The resulting crude brain capillaries-rich pellets were resuspended in MIB and filtered through two nylon filters of 120- and 30-μm mesh size (Millipore). The quality of trapped brain capillaries in 30-μm filters was checked. Brain capillaries were stored at −80°C until further use.

### Protein extraction

For total protein analysis isolated brain capillaries were homogenized and lysed in NP-40 lysis buffer supplemented with 5% Protease Inhibitor Cocktail and 1% Phosphatase Inhibitor Cocktail 2. Lysate samples were sonicated over two cycles lasting 20 s each at 4°C at 40% power. For nuclear protein analysis, nuclear fraction was extracted using commercially available kit (Thermo Scientific, IL, USA). Protein concentrations were measured by means of the Quantipro BCA assay kit (Sigma-Aldrich) according to the manufacturer’s protocol.

### Western blot analysis

For total and phosphorylated protein analyses, 2× SDS loading buffer was added to lysate samples containing equal amounts of protein (10 μg). These samples were heated for all protein analysis studies except for those involving ABCB1, for which samples were loaded without heating to avoid aggregation of these highly glycosylated transmembrane proteins. Samples were subjected to SDS–polyacrylamide gel electrophoresis (SDS-PAGE) followed by Western blot analysis, with primary antibodies diluted 1:1000 in 5% skim milk (Sigma-Aldrich) and 0.1 M tris-buffered saline–Triton X-100 (TBS-T). The following antibodies were used: Anti-ABCB1 (sc-8313), and -Lamin B (sc-6217) were purchased from Santa Cruz Biotechnology. Anti total GSK3α/β (5676), and total β-catenin (9562) were purchased from Cell Signaling Technology. Anti tyrosine phosphorylated GSK3α/β (ab4797) was obtained from Abcam, anti occludin (71–1500) was purchased from Invitrogen, and anti β-actin (MAB1501) was purchased from Chemicon. Primary antibodies were detected with horseradish peroxidase (HRP)–conjugated secondary immunoglobulin G (IgG) that were diluted 1:5000 in 5% skim milk and TBS-T and revealed by enhanced chemiluminescence plus (ECL) solution (Amersham International). Blots were digitized, densitometrically analyzed with ImageJ image analysis software (NIH), corrected for protein loading by means of Lamin B (nuclear β-catenin), or β-actin (all others) blots, and expressed as relative values comparing ipsilateral (hypoperfused) with contralateral (non hypoperfused).

### Statistics

Results are expressed as mean ± standard error of the mean (SEM). For multiple comparisons statistical analysis was performed using the one-way analysis of variance (ANOVA), followed by Tukey’s post hoc tests. For the comparison between two groups data were analyzed using standard two-tailed unpaired t-test's. A P value < 0.05 was considered statistically significant. All analyses were performed using GraphPad Prism Version 6 for Windows (GraphPad Software, San Diego, CA, USA).

## Results

### Hypoperfusion induces neurovascular dysfunction

**Figure 1:**
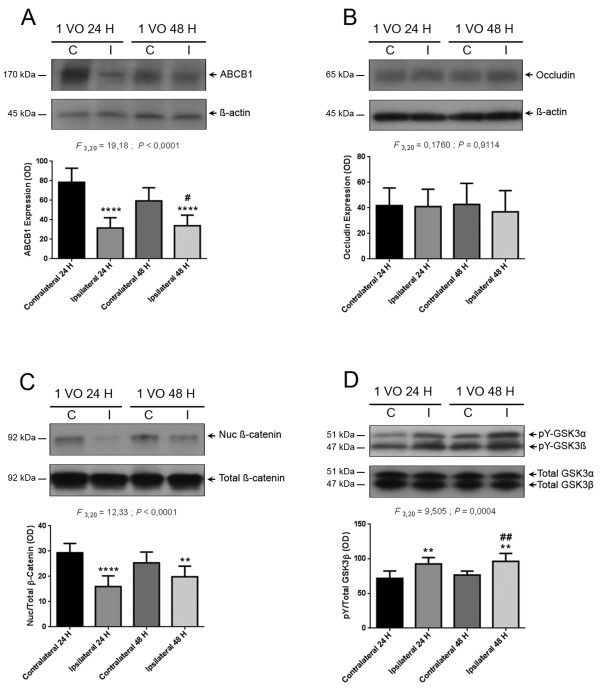
**Hypoperfusion decreases ABCB1 protein levels in brain capillaries through GSK3β/β-catenin pathway.** Western blot analysis using brain capillary extracts showing that 24 and 48 hours after 1VO, **(A)** ABCB1 protein levels are decreased, **(B)** occludin protein expression is unchanged, **(C)** β-catenin nuclear abundance is decreased, and **(D)** tyrosine phosphorylation (pY) of GSK3β is increased, in ipsilateral hemisphere. Data are means ± SEM (n = 6). C, contralateral non-hypoperfused brain capillaries; I, ipsilateral hypoperfused brain capillaries; 1 VO 24 H, one vessel occlusion for 24 hours; 1 VO 48 H, one vessel occlusion for 48 hours. **P < 0.01 / ****P < 0.0001 compared with contralateral 24 hours // ^##^P < 0.01 compared with contralateral 48 hours (One-way analysis of variance (ANOVA) followed by Tukey’s multiple comparisons test).

ABCB1 is considered to be a marker of NVU functionality, whereas the tight junction protein occludin is a marker of NVU physical integrity [15]. As such, we investigated the effect of mild chronic cerebral hypoperfusion, on the functional and physical properties of the NVU, by analyzing the protein expression of ABCB1 and occludin in brain capillaries. Interestingly, a robust decrease in ABCB1 protein levels was observed 24 hours after 1 VO in ipsilateral (i.e. hypoperfused), but not in contralateral (i.e. non hypoperfused) brain capillaries (Figure 1A), which persisted 48 hours after 1 VO (Figure 1A). In contrast to ABCB1, occludin protein levels did not change in both hemispheres (Figure 1B).

### Hypoperfusion regulates GSK3β/β-catenin signaling pathway in brain capillaries

The nuclear translocation of β-catenin plays a central role in inducing NVU functionality, mainly by increasing ABCB1 gene expression and protein levels [25]. β-catenin abundance in the cytosol and its subsequent translocation to the nucleus are tightly controlled by the kinase activity of GSK3β that serine phosphorylates β-catenin, thus triggering its proteasomal degradation [15]. Both proteins constitute major components of the Wnt signaling pathway [25]. The activity of GSK3β is controlled by two phosphorylation states, the serine phosphorylation that induces its deactivation, and the tyrosine phosphorylation that enhances its kinase activity [26]. To decipher the molecular mechanisms involved in NVU dysfunction translated by ABCB1 decreased expression upon hypoperfusion, we analyzed the nuclear levels of β-catenin, and tyrosine phosphorylation state of GSK3β in brain capillaries. Mild chronic cerebral hypoperfusion significantly decreased nuclear β-catenin levels 24 and 48 hours after 1 VO in ipsilateral brain capillaries (Figure 1C), which was accompanied by an increased tyrosine phosphorylation (i.e. enhanced activation) of GSK3β (Figure 1D).

### Peripherally applied human Aβ_1-42_ peptides form small deposits in hypoperfused brain capillaries

**Figure 2:**
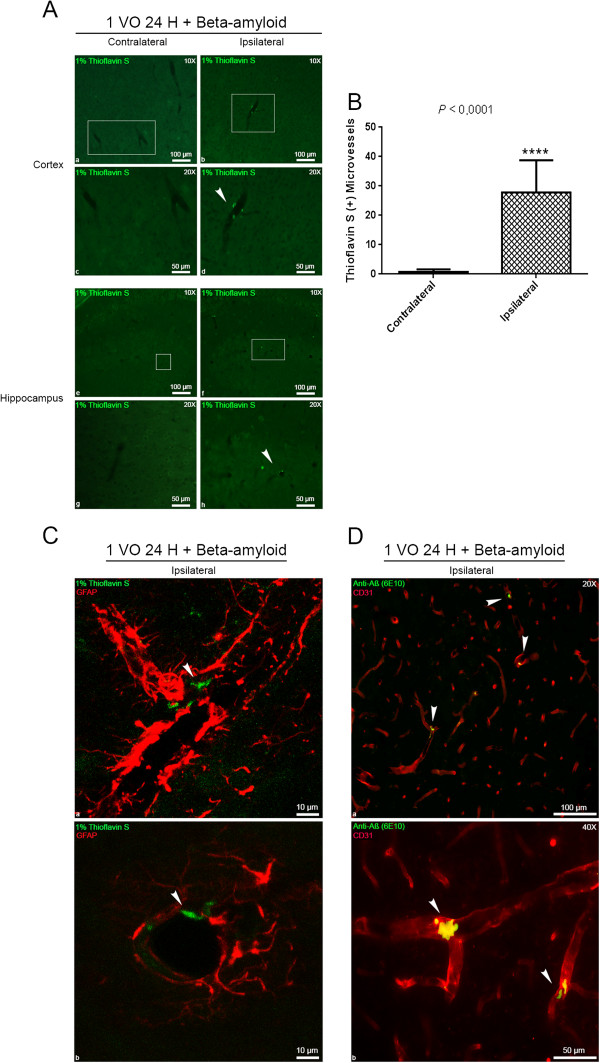
**Hypoperfusion triggers vascular deposition of peripherally applied human Aβ**_**1-42 **_**peptides.** 1% Thioflavin S **(A)**, combined with immunofluorescence **(C)** and double immunofluorescence **(D)** analysis examining the deposition of Aβ aggregates, and their sub-cellular localization in brain capillaries 24 hours after Aβ_1-42_ administration in mice that were hypoperfused for 24 hours. **(A, B)** Thioflavin S positive Aβ deposits are observed in ipsilateral brain capillaries of the cortex and hippocampus, and are almost absent in contralateral brain capillaries of the same animals, and in the brain capillaries of saline treated animals. **(C)** Thioflavin S positive Aβ deposits are localized in the perivascular space of brain capillaries within GFAP positive astrocytic endfeet. **(D)** Endothelial marker CD31, and Anti human 6E10 Aβ double staining confirm the vascular localization of Aβ deposits in ipsilateral brain capillaries. Data are means ± SEM (n = 5 animals per group, 5 sections per animal’s brain). Arrowheads show vascular Thioflavin S/6E10 positive aggregates.1 VO 24 H, one vessel occlusion for 24 hours. ****P < 0.0001 compared with contralateral hemisphere (two-tailed unpaired t-test's).

Mild chronic cerebral hypoperfusion has been proposed to play a critical role in the pathogenesis of AD [8]. More precisely, cerebral hypoperfusion has been suggested to precede and contribute to the onset of AD clinical manifestations [14]. In order to understand the role of mild chronic cerebral hypoperfusion in time-related Aβ deposition, we investigated the fate of peripherally applied human Aβ_1-42_ peptides (25 ng; the equivalent quantity found in one week in the serum of littermate APP/PS1 transgenic mouse) in the brain capillaries of hypoperfused (24 hours after 1 VO) mice. Interestingly, we detected Thioflavin S positive Aβ deposits, predominantly in ipsilateral brain capillaries in both cortex, and hippocampus 24 hours after human Aβ_1-42_ intravenous injection (Figure 2A). The total count of Thioflavin S positive brain capillaries was significantly higher in the ipsilateral hemisphere compared to the contralateral hemisphere (Figure 2B). Interestingly, Thioflavin S positive Aβ deposits were found at the abluminal side of ipsilateral brain capillaries between astrocyte endfeet as soon as 24 hours after injection (Figure 2C). Moreover, the human specific anti-Aβ (6E10) antibody confirmed Thioflavin S positive Aβ deposits that were detected in ipsilateral brain capillaries (Figure 2D).

### Aβ deposits shift from highly vascular to the parenchyma six weeks after hypoperfusion

**Figure 3:**
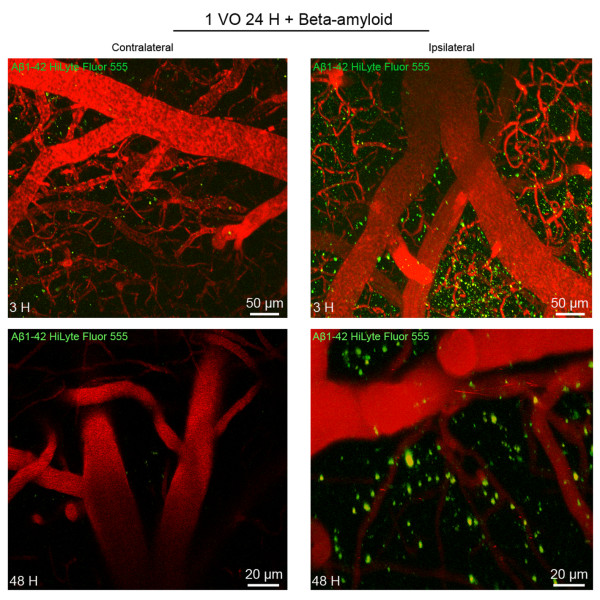
**Peripherally applied human Aβ**_**1-42 **_**peptides enter the hypoperfused hemisphere.** Stacked images obtained from two-photon intravital imaging examining brain distribution of peripherally applied human Aβ_1-42_ Hilyte Fluor 555 (10 μg), 3 hours, and 48 hours after intravenous injection of 24 hours hypoperfused mice. Aβ_1-42_ Hilyte Fluor 555 is detected in large quantities in the ipsilateral hemisphere around brain capillaries after injection, but the signal was barely detectable in the contralateral hemisphere at 3 and 48 hours. The same mouse was used during the time course (n = 2). 1 VO 24 H, one vessel occlusion for 24 hours.

**Figure 4:**
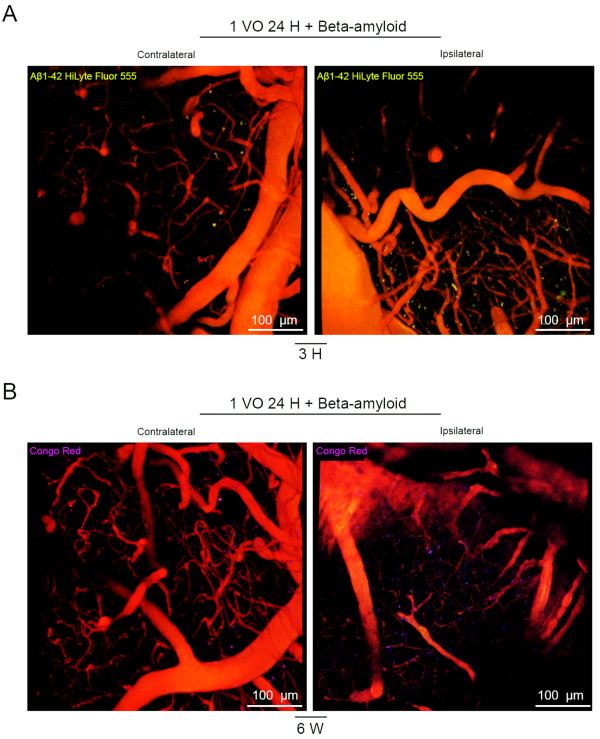
**Peripherally applied human Aβ**_**1-42 **_**peptides form small aggregates in the hypoperfused hemisphere.** Stacked images obtained from two-photon intravital imaging examining brain distribution and deposition of peripherally applied human Aβ_1-42_ Hilyte Fluor 555 (25 ng), 3 hours **(A)**, and 6 weeks **(B)** after intravenous injection of 24 hours hypoperfused mice. **(A)** Aβ_1-42_ Hilyte Fluor 555 is detected mostly around ipsilateral brain capillaries 3 hours after injection. **(B)** Congo Red positive Aβ aggregates are exclusively detected in the hypoperfused hemisphere. Two video sections were generated from these stacks (Additional file 3: Video S1 = Ipsiateral hemisphere; Additional file 4: Video S2 = Contralateral hemisphere). The same mouse was used during the time course (n = 2). 1 VO 24 H, one vessel occlusion for 24 hours.

To follow up the kinetics of peripherally applied human Aβ_1-42_ peptides in entering the brain, we evaluated the time course and biodistribution of Aβ *in vivo* (Additional file 2: Figure S2). In a first set of experiments, 24 hours after 1 VO, mice received a single intravenous injection of human Aβ_1-42_ Hilyte Fluor 555 (10 μg), and Aβ was tracked using intravital microscopy in the same animals *in vivo*, 3 hours, and 48 hours after injection. The peripherally applied human Aβ_1-42_ peptides massively entered the ipsilateral hemisphere as early as 3 hours after injection, and almost no Aβ was detected in the contralateral side (Figure 3). Interestingly, human Aβ_1-42_ peptides seemed highly associated to brain capillaries 3 hours after injection, and began to be associated to brain parenchyma 48 hours post-injection (Figure 3). In a second set of experiments, mice received a single intravenous injection of human Aβ_1-42_ Hilyte Fluor 555 (25 ng) 24 hours after 1 VO and was followed by intravital microscopy in the same animals *in vivo*. Similar to the previous set of experiments, massive entry of Aβ was observed in the ispilateral hemisphere as early as 3 hours after injection, and almost no Aβ was detected in the contralateral side (Figure 4A). In order to visualize and investigate Aβ deposition and possible aggregation in the brain, Congo Red dye was injected in the Cisterna Magna (Additional file 2: Figure S2). Interestingly a single systemic injection of human Aβ_1-42_ (25 ng) was sufficient to seed small Aβ aggregates in the ipsilateral parenchymal hemisphere 6 weeks later (Figure 4B), highlighted in an additional movie file that shows a 3-dimensional illustration of the ipsilateral hemisphere (Additional file 3: Video S1). It is noteworthy that almost no deposits were found in the contralateral side (Figure 4B), highlighted in an additional movie file that shows a 3-dimensional illustration of the contralateral hemisphere (Additional file 4: Video S2).

### Hypoperfusion induces NVU dysfunction by altering brain perfusion and glucose metabolism

**Figure 5:**
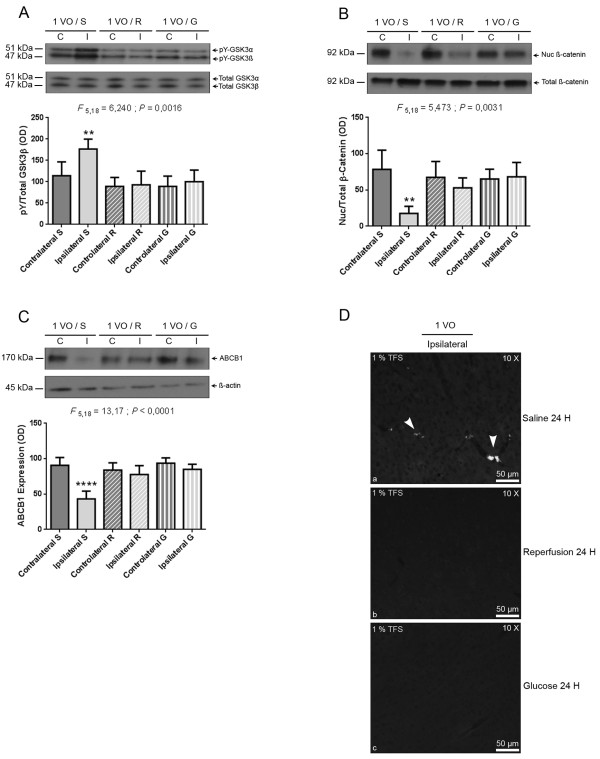
**Brain reperfusion, or glucose administration re-established ABCB1 protein levels in hypoperfused brain capillaries.** Western blot analysis using brain capillary extracts shows that 24 after brain reperfusion, or glucose administration in 24 hours hypoperfused mice, **(A)** tyrosine phosphorylation (pY) of GSK3β is decreased, **(B)** β-catenin nuclear abundance is rescued, and **(C)** ABCB1 protein returned to normal levels in the ipsilateral hemisphere. **(D)** 1% Thioflavin S staining is not detected in the hypoperfused brain capillaries 24 hours after brain reperfusion, or glucose administration in 24 hours hypoperfused mice. Data are means ± SEM (n = 4). C, contralateral non-hypoperfused brain capillaries; I, ipsilateral hypoperfused brain capillaries; 1 VO, one vessel occlusion for 24 hours; S, saline; R, brain reperfusion; G, glucose administration. **P < 0.01 / ****P < 0.0001 compared with contralateral saline (One-way analysis of variance (ANOVA) followed by Tukey’s multiple comparisons test).

**Figure 6:**
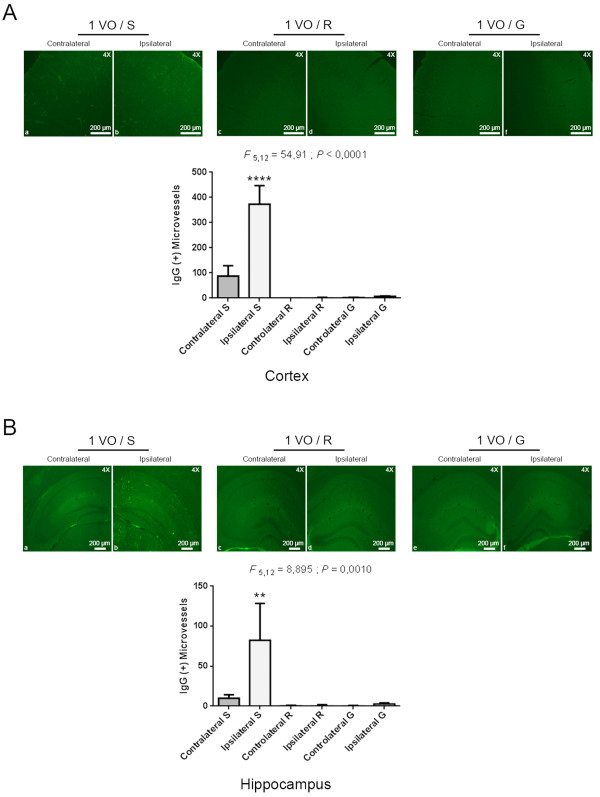
**Brain reperfusion, or glucose administration decreased IgG entrapment in hypoperfused brain capillaries.** Immunofluorescence analysis examining endogenous IgG entrapment in brain capillaries shows that brain reperfusion, or glucose administration decreases IgG entrapment in the brain capillaries of the cortex **(A)**, and similarly decreases IgG entrapment in the brain capillaries of the hippocampus **(B)**. Data are means ± SEM (n = 3 animal per group, 3 sections per animal’s brain). 1 VO, one vessel occlusion for 24 hours; S, saline; R, brain reperfusion; G, glucose administration. **P < 0.01 / ****P < 0.0001 compared with contralateral saline (One-way analysis of variance (ANOVA) followed by Tukey’s multiple comparisons test).

GSK3β plays an important role in glucose metabolism and glucose deprivation has been shown to activate GSK3β [13]. CBF perturbations and glucose metabolism deregulation have been previously described in AD [11,12]. In line with these observations, we investigated the role of brain perfusion and glucose in inducing NVU dysfunction and Aβ deposition cascade in ipsilateral brain capillaries. Interestingly, both reperfusion and glucose administration significantly reduced the tyrosine phosphorylation of GSK3β (Figure 5A), rescued nuclear β-catenin levels (Figure 5B) and reestablished the basal expression of ABCB1 (Figure 5C) in ipsilateral brain capillaries. In another set of experiments, rCCA reperfusion and the intravenous injection of glucose were accompanied by the intravenous injection of human Aβ_1-42_ (25 ng). Interestingly, both reperfusion and glucose administration abolished the early Aβ vascular deposition (Figure 5D). We next evaluated Immunoglobulin G (IgG) entrapment inside these capillaries and no signal was found in the cortex (Figure 6A) and the hippocampus (Figure 6B).

## Discussion

By using a mouse model of mild chronic cerebral hypoperfusion, we show that ABCB1 protein levels in brain capillaries were significantly decreased 24 hours after 1 VO. Such a phenomenon does not depend on the integrity of the NVU since expression of the tight junction protein occludin remained unchanged. Alteration of ABCB1 seems to be the consequence of a decreased nuclear translocation of β-catenin, which was caused by the enhanced activation of GSK3β in hypoperfused brain capillaries. This observation was due to a deregulation in glucose metabolism in ipsilateral brain capillaries due to an inadequate blood supply. Indeed, brain reperfusion and the administration of high dose of glucose inhibited GSK3β activation, re-established β-catenin nuclear translocation, and rescued ABCB1 protein levels. Mild chronic cerebral hypoperfusion caused a general dysfunction in brain capillaries, which was characterized by exacerbated IgG entrapment in these capillaries. Thioflavin S positive Aβ small deposits were also exclusively detected in ipsilateral brain capillaries and within astrocyte endfeet 24 hours after injection of human Aβ_1-42_ peptides in hypoperfused mice. The presence of Thioflavin S positive Aβ deposits within astrocyte endfeet confirms the passage of Aβ from the blood into the perivascular space, where they aggregate due a faulty clearance. Six weeks later, Aβ vascular deposits were seeded in brain parenchyma as small Aβ Congo Red positive aggregates, which were detected by intravital microscopy in the brain of living mice. Interestingly, brain reperfusion or the administration of high dose of glucose prevented Aβ deposition in ipsilateral brain capillaries. Taken together these data indicate that mild chronic cerebral hypoperfusion directly contributes in the initiation of Aβ deposition and aggregation cascades. Our study supports recent data showing that cerebral hypoperfusion accelerates cerebral amyloid angiopathy (CAA) pathology in humans and CAA mice model, by deeply affecting CBF and neurovascular function [27].

Still today, despite the considerable efforts, the initiation of neurodegenerative cascades in AD remains unknown. The difficulty is that the disease is diagnosed mainly at the late stages where the pathology is irreversibly installed. Cerebral hypoperfusion has been suggested to play an important role at the early stages of AD pathogenesis and have been proposed to precede and contribute to early-clinical onset of AD [14]. The two-hit vascular hypothesis of AD speculates that the NVU dysfunction and mild chronic cerebral hypoperfusion contribute to the initiation of AD pathogenesis, which leads afterwards to Aβ deposition and aggregation into the brain, and consequently tau protein hyperphosphorylation [8]. Indeed, NVU dysfunction has been reported in AD [28], and the key marker of NVU functionality that is actively involved in the clearance of Aβ peptides, is ABCB1 [17]. ABCB1 protein levels and transport activity are reduced in the brain capillaries of mouse models of AD [29]. In parallel, the clearance of intracerebrally injected Aβ peptides is impaired in the brain of mice lacking ABCB1 [30]. Similarly, ABCB1 levels are decreased in the brain capillaries surrounding amyloid plaques in post-mortem brain samples of AD patients [18]. Interestingly, ABCB1 levels are undetectable in the brain capillaries of post-mortem brain samples of CAA patients [31]. Taken together, these data outline the link between ABCB1 expression, function and Aβ peptides processing, and clearance.

It is not possible to decipher the mechanisms involved in these phenomena, because these data were generated at late stages of AD pathogenesis. With our mouse model that excludes several artificial interfering factors, we show that mild chronic cerebral hypoperfusion deeply affected NVU function by causing a sustained decrease in ABCB1 protein levels in ipsilateral brain capillaries, via a mechanism involving GSK3β/β-catenin pathway. Nuclear β-catenin translocation induces the activation of transcription factors belonging to the Lymphoid Enhancer Factor (LEF)/T cell factor (TCF) family that controls abcb1 gene expression [25]. β-catenin nuclear abundance is tightly controlled by the kinase GSK3β [25]. Once activated, GSK3β mediates β-catenin serine phosphorylation, thus triggering its proteasomal degradation [15]. We show here that mild chronic cerebral hypoperfusion induces GSK3β activation via tyrosine phosphorylation, leading to β-catenin degradation.

Adequate brain perfusion is critical for a proper glucose biodistribution. Several studies have reported glucose metabolism deregulation in AD, most probably as a consequence of CBF irregularities [11,12]. GSK3β has been widely investigated in AD and is the target of several pharmacological compounds aiming to inhibit its kinase activity [32]. GSK3β is constitutively active and plays a key role in glucose metabolism [33]. However, GSK3β activity is triggered when glucose levels are not optimal. Such an activation is caused by either decreased serine phosphorylation or enhanced tyrosine phosphorylation, which lead to Glycogen Synthase (GS) inhibition, an enzyme that converts the excess of glucose into glycogen [34]. Mild chronic cerebral hypoperfusion affected glucose metabolism, most probably by impairing glucose biodistribution leading to GSK3β activation. Indeed, we show here that brain reperfusion or glucose administration abolished the effects of mild chronic cerebral hypoperfusion on NVU functionality, evidenced by basal GSK3β tyrosine phosphorylation, nuclear β-catenin abundance and ABCB1 protein levels in brain capillaries.

**Figure 7:**
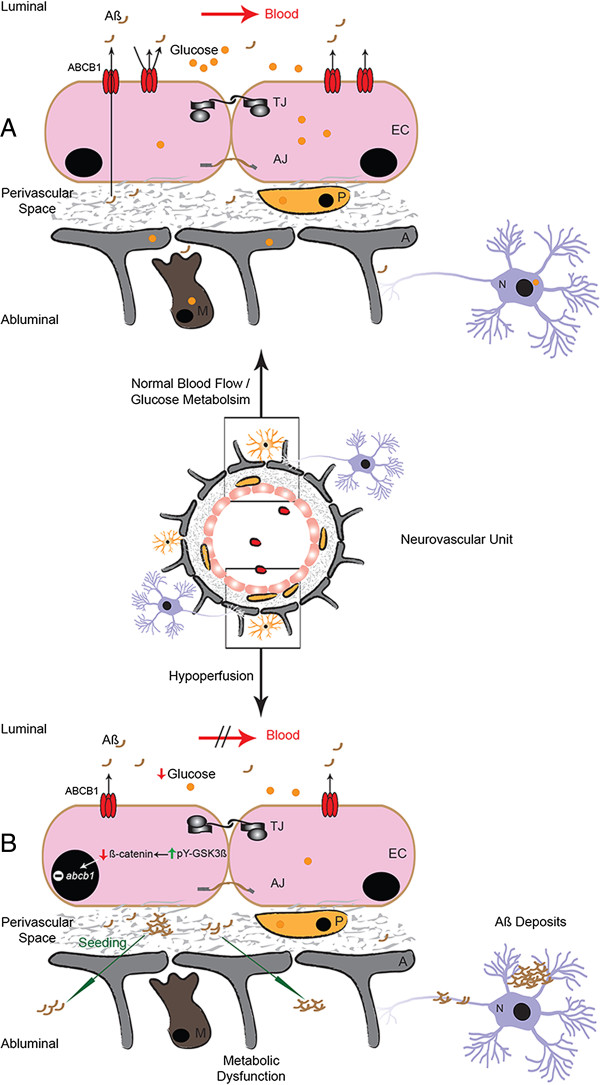
**Mild chronic cerebral hypoperfusion initiates Aβ deposition and aggregation.** Cerebral hypoperfusion deregulates glucose supply to the brain, and creates a metabolically distressed microenvironment within the NVU. The metabolic stress triggers NVU dysfunction by enhancing the activation of GSK3β, via tyrosine phosphorylation (pY), in an attempt from the brain to preserve glucose metabolism. GSK3β enhanced activity leads to an excessive degradation of β-catenin, therefore reduces its nuclear abundance, which consequently decreases ABCB1 protein levels in brain capillaries. Under these conditions the NVU is unable to fulfill its role in protecting the brain from Aβ entry and accumulation. Consequently, Aβ peptides massively enter the brain and form small aggregates in the perivascular space of hypoperfused brain capillaries. Aβ vascular deposits shift with the time to brain parenchyma, seeding small Aβ aggregates, thus initiating the neurodegenerative cascades of AD. EC, Endothelial Cells; P, Pericytes; A, Astrocyte Endfeet; TJ, Tight Junctions; AJ, Adherent Junctions.

The amyloid hypothesis suggests that the excessive accumulation of cerebral Aβ, which is the outcome of an imbalanced Aβ production and clearance, is central in initiating the disease, and triggering tau tangle formation [3]. With time, soluble Aβ begins to accumulate in the brain to from insoluble aggregates that can evolve to form amyloid plaques [1]. Increased blood levels of Aβ have been reported in AD patients at the early stages of the disease [35]. These observations suggested that Aβ equilibrium between the blood and brain is a critical mechanism where the NVU plays a key role [8]. NVU dysfunction caused by a faulty cerebral perfusion interferes with such equilibrium leading to the initiation of Aβ deposition cascade [8]. Here we show that mild chronic cerebral hypoperfusion induced NVU dysfunction and created a metabolically distressed microenvironment that initiated and facilitated the vascular deposition of systemically applied human Aβ_1-42_ into small aggregates, which have penetrated the ipsilateral brain parenchyma in a relatively short period (Figure 7). Our observation is in line with recent report showing that cerebral hypoperfusion increased Aβ vascular deposition by impairing ISF-mediated Aβ drainage along the perivascular space of brain arteries in a mouse model of AD [36]. More importantly, in the same study the authors suggested the existence of a feed-forward mechanism, by which amyloid deposition promotes further amyloid deposition. The implication of an impaired ISF-mediated Aβ drainage along the perivascular space of brain arteries in our animal model is highly probable, which would amplify the impact of ABCB1 reduction, therefore further increasing the accumulation Aβ peptides in the brain. Inoculating synthetic Aβ peptides in mice via many different routes have been attempted to investigate their entry and deposition into the brain, without success [37]. On the other hand, inoculating Aβ rich brain extracts of APP transgenic mice in young APP transgenic mice succeeded in inducing cerebral β-amyloidosis [38,39]. Taken together, these observations suggest a prion-like behavior of Aβ rich brain extracts of APP transgenic mice may be a prerequisite to induce cerebral β-amyloidosis. In this regard our study suggests a crucial role of mild chronic hypoperfusion in creating such a suitable microenvironment to drive the aggregation of synthetic Aβ_1-42_.

## Conclusions

These findings demonstrate the central role of mild chronic cerebral hypoperfusion in inducing NVU dysfunction, which was sufficient to induce rapid Aβ deposition in brain capillaries in response to systemically-administered Aβ_1-42_ peptides. Such a selective accumulation in capillaries of the ipsilateral side is followed by Aβ deposition, aggregation, and seeding in the brain. Moreover, these results highlight the importance of mild chronic cerebral hypoperfusion in inducing the aggregation of Aβ_1-42_ peptides. It would be very interesting to check tau protein phosphorylation in this model, which is something we are actually perusing. These data may underline the early events that take place in the initiation of AD pathogenesis in a new mouse model without amyloid gene-associated transgenes, which is crucial for developing preventive strategies, by acting at the early stages of the disease.

## Abbreviations

NVU: Neurovascular unit; CBF: Cerebral blood flow; ISF: Interstitial fluid; BBB: Blood–brain barrier; rCCA: Right common carotid artery; GSK3β: Glycogen synthase kinase 3; APP: Amyloid precursor protein; ECM: Extracellular matrix; ABC transporter: ATP-Binding cassette transporter; CAA: Cerebral amyloid angiopathy; LEF/ TCF: Lymphoid enhancer factor/T cell factor TCF; GS: Glycogen synthase.

## Competing interests

The authors declare that they have no competing interests.

## Authors’ contributions

AEA and SR conceived and designed the study, and drafted the manuscript. AEA and PT performed the experiments, analyzed and interpreted data. PP acquired the two-photon intravital microscopy images. All authors read and approved the final manuscript.

## Authors’ information

SR is a Full Professor and Principal Investigator at the Department of Molecular Medicine, Faculty of Medicine, University of Laval, and the Director of CHU de Québec Research Center (CHUL). *Correspondence: Email: serge.rivest@crchul.ulaval.ca.

## Supplementary Material

Additional file 1: Figure S1 Schematic representation of the protocols used in the study. A scheme illustrating the different protocols used to perform this study. Details on operations, sacrifice time points, and the experimental procedures applied for each protocol are provided. A total number of 100 mice were used in this study. The number of animals used in each experimental procedure are mentioned in figure legends. Click here for file

Additional file 2: Figure S2 Schematic representation of the two-photon intravital experimental procedure. An incision was made to expose the skull and two small cranial windows are drilled corresponding to the following coordinates: (i) A/P +0.83 mm, M/L +0.5 mm, and A/P +0.83 mm, M/L -0.5 mm relative to the bre gma. Congo Red solution is injected in the Cisterna Magna, and human soluble monomer Aβ_1-42_ Hilyte Fluor 555 is injected intravenously via the tail vein. Click here for file

Additional file 3 **Video S1.** Animated 3-dimensional illustration of Figure 4B ispilateral hemisphere. An animated 3-dimensional illustration showing the presence of Congo Red positive aggregates (purple) within the structure of the ipsilateral hemisphere. The 3-dimensional illustration was generated by processing, in ImageJ software, 101 images (stacks) that were acquired by two-photon intravital microscopy at a depth ranging from 50 μm (i.e. cortex surface) to 250 μm (inside the cortex), for a total depth of 200 μm. Click here for file

Additional file 4 **Video S2.** Animated 3-dimensional illustration of Figure 4B contralateral hemisphere. An animated 3-dimensional illustration showing the absence of Congo Red positive aggregates within the structure of the contralateral hemisphere. The 3-dimensional illustration was generated by processing, in ImageJ software, 101 images (stacks) that were acquired by two-photon intravital microscopy at a depth ranging from 50 μm (i.e. cortex surface) to 250 μm (inside the cortex), for a total depth of 200 μm. Click here for file
