# Supplementary material for: Mild chronic cerebral hypoperfusion induces neurovascular dysfunction, triggering peripheral beta-amyloid brain entry and aggregation
Source: Acta Neuropathol Commun. 2013 Nov 13;1:75. doi: 10.1186/2051-5960-1-75 (PMC3843528; doi:10.1186/2051-5960-1-75)
Supplement: Additional file 1: Figure S1 — Schematic representation of the protocols used in the study. A scheme illustrating the different protocols used to perform this study. Details on operations, sacrifice time points, and the experimental procedures applied for each protocol are provided. A total number of 100 mice were used in this study. The number of animals used in each experimental procedure are mentioned in figure legends. [file 2051-5960-1-75-S1.doc]

**
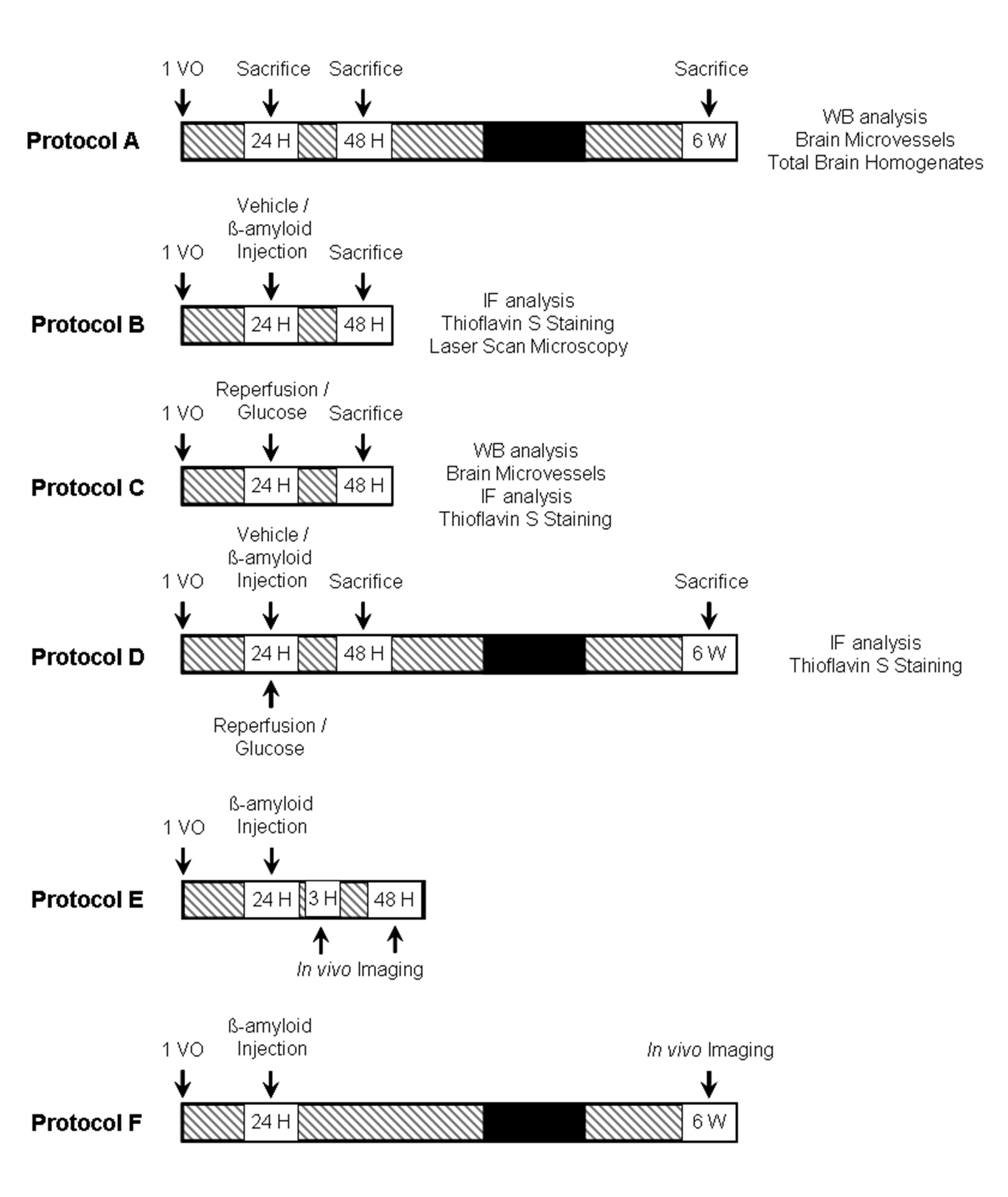
**

**Figure S1.** **Schematic representation of the protocols used in the study**. A scheme illustrating the different protocols used to perform this study. Details on operations, sacrifice time points, and the experimental procedures applied for each protocol are provided. A total number of 100 mice were used in this study. The number of animals used in each experimental procedure are mentioned in figure legends.
