# Supplementary material for: Mild chronic cerebral hypoperfusion induces neurovascular dysfunction, triggering peripheral beta-amyloid brain entry and aggregation
Source: Acta Neuropathol Commun. 2013 Nov 13;1:75. doi: 10.1186/2051-5960-1-75 (PMC3843528; doi:10.1186/2051-5960-1-75)
Supplement: Additional file 2: Figure S2 — Schematic representation of the two-photon intravital experimental procedure. An incision was made to expose the skull and two small cranial windows are drilled corresponding to the following coordinates: (i) A/P +0.83 mm, M/L +0.5 mm, and A/P +0.83 mm, M/L -0.5 mm relative to the bre gma. Congo Red solution is injected in the Cisterna Magna, and human soluble monomer Aβ1-42 Hilyte Fluor 555 is injected intravenously via the tail vein. [file 2051-5960-1-75-S2.doc]

**
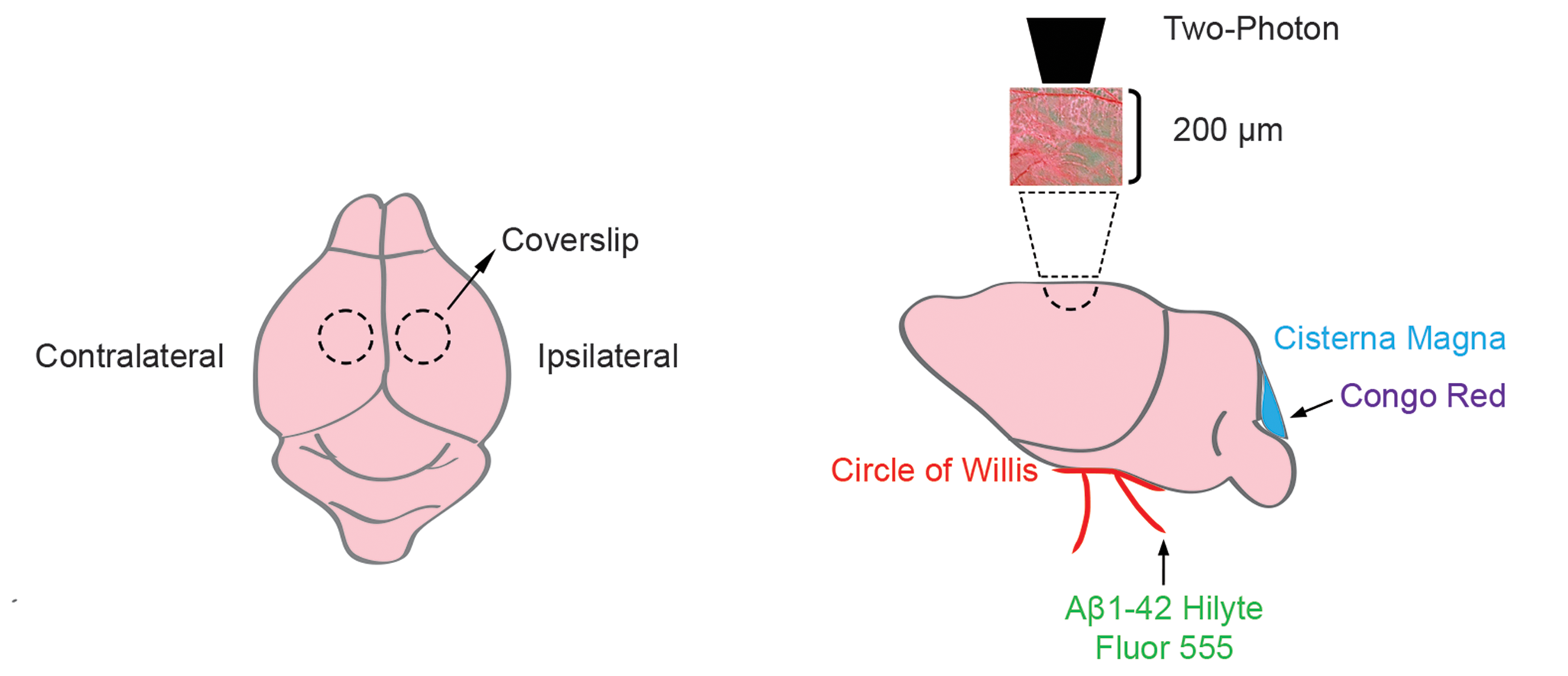
**

**FigureS2.** **Schematic representation of the two-photon intravital experimental procedure.** An incision was made to expose the skull and two small cranial windows are drilled corresponding to the following coordinates: (i) A/P +0.83 mm, M/L +0.5 mm, and A/P +0.83 mm, M/L -0.5 mm relative to the bre gma. Congo Red solution is injected in the Cisterna Magna, and human soluble monomer Aβ_1-42_ Hilyte Fluor 555 is injected intravenously via the tail vein.
